# The impact of physical activity on executive function in college students: the chain mediating role of anxiety and sleep quality

**DOI:** 10.3389/fpsyg.2026.1853278

**Published:** 2026-07-29

**Authors:** Yuhao Li, Xiaotong Wan, Aojie Ru, Yanfei Wang, Jing Wang, Yang Liu, Zhiru Liang, Hui Zhang, Yin Bao

**Affiliations:** 1 Beijing Sport University, Beijing, China; 2 School of Nursing, Anhui Medical University, Hefei, China; 3 Department of Science and Education, Xilingol League Central Hospital, Xilinhot, China; 4 Department of Anesthesiology for Surgery, Xilingol League Central Hospital, Xilinhot, China; 5 Jishou University, Jishou, China; 6 Department of Hepatobiliary Surgery, Xilingol League Central Hospital, Xilinhot, China; 7 Department of Nursing, The First Affiliated Hospital of Anhui Medical University (Anhui Public Health Clinical Center), Hefei, China

**Keywords:** anxiety, chain mediation, college students, executive function, physical activity, sleep quality

## Abstract

**Background and objectives:**

Executive function (EF) serves as a critical regulatory hub for goal-directed behavior, yet its operational integrity is increasingly threatened by the sedentary lifestyles and psychological stressors prevalent in higher education. While the cognitive benefits of exercise are documented, the specific pathways through which it navigates psychological and physiological barriers remain underexplored. This study constructs a chain mediation model to elucidate how physical activity (PA) optimizes EF by sequentially modulating anxiety and sleep quality (SQ) among university students.

**Methods:**

Data collection was conducted in December 2025, involving a multi-stage cluster convenience sample of 2,033 students across six Chinese provinces. Participants (mean age 19.0 ± 1.1) completed a battery of validated instruments, including the Single-Item Measure for PA, the GAD-2 for anxiety, the Single-Item Sleep Quality Scale, and the Adolescent Executive Function Scale. Structural relationships were analyzed using the SPSS PROCESS macro (Model 6) while controlling for demographic covariates such as gender, age, and socioeconomic background.

**Results:**

Path analysis confirmed that PA positively predicts EF proficiency, with the direct effect accounting for 49.53% of the total variance. Beyond this direct link, the relationship is substantiated by three distinct mediating trajectories: (1) a psychological pathway via anxiety reduction (31.78%), (2) a physiological pathway via enhanced sleep quality (14.02%), (3) a sequential chain pathway wherein PA alleviates anxiety, which subsequently optimizes sleep, leading to superior executive performance (4.67%). All mediating effects reached statistical significance as evidenced by bootstrap confidence intervals.

**Conclusion:**

These findings transition the understanding of physical intervention from a unilinear correlation to a multi-systemic synergistic response. Exercise functions not only as a cognitive catalyst but also as a vital buffer that prevents the maladaptive depletion of neural resources by stabilizing emotional oscillations and facilitating physiological restoration. The results underscore the necessity for higher education institutions to integrate physical activity, psychological counseling, and sleep hygiene management into a cohesive framework for cognitive health.

## 1 Background

Executive function (EF) represents a multifaceted constellation of high-level cognitive processes rather than a singular mental operation. It serves as a regulatory hub that coordinates and optimizes the execution of complex goal-directed tasks, thereby ensuring behavioral efficacy (Huang et al., 2014). This intricate architecture is underpinned by three core components: inhibitory control, working memory, and cognitive flexibility. Although these sub-domains operate through distinct mechanisms, they are functionally integrated to facilitate intelligent behavior. Cognitive flexibility, in particular, enables individuals to deliberate prior to action, allowing for the fluid recalibration of strategies in response to environmental shifts (Banich, 2009; Diamond, 2013). Furthermore, it assists in transcending cognitive rigidity, fostering multi-perspective approaches to novel challenges. Consequently, EF proficiency is a robust predictor of long-term cognitive trajectory, social maturation, and academic attainment. However, impairments or developmental delays in this command system can trigger a cascade of adverse outcomes, ranging from localized learning difficulties to broader neurodevelopmental and psychiatric crises (Banich, 2009; Zelazo et al., 2003). The operational integrity of EF is subject to the dual constraints of physiological and psychological factors. Modern lifestyle stressors—specifically sedentary behavior and deteriorating sleep quality—directly compromise both transient neural performance and long-term brain development. From a psychological standpoint, negative emotional interference is a critical determinant of EF efficiency. Chronic stress and anxiety do more than induce subjective distress; they preemptively consume finite cognitive resources, leading to performance degradation upon resource depletion (Carlson et al., 2013; Diamond and Lee, 2011). This phenomenon is especially pronounced in contemporary higher education settings. As physical inactivity and sleep deprivation become endemic among university students (Jiazhi et al., 2025), the resulting emotional strain and perceptual fatigue have become pervasive. Given the critical role of EF in fostering academic resilience, exploring whether physical activity can mitigate cognitive attrition by enhancing psycho-physiological states is essential. Such investigation not only refines existing cognitive frameworks but also provides practical pathways for optimizing the psychological development of the collegiate population.

Physical activity (PA), defined as the process of generating force through skeletal muscle activation, encompasses diverse modalities ranging from fundamental aerobic exercise to specialized endurance and coordination training (Knuttgen, 2003). Beyond its established role in optimizing physical health and motor proficiency, contemporary research underscores PA as a potent catalyst for cognitive development. Nevertheless, a stark discrepancy persists between theoretical consensus and behavioral reality: while the cognitive benefits of exercise are well-documented, sedentary behavior among university students has reached alarming levels. Recent data from Chinese higher education institutions indicate that approximately 89.0% of students exhibit physical inactivity (Han et al., 2025). This exercise deficit poses a subtle yet persistent threat to cognitive integrity. Evidence suggests that EF can be substantially augmented through both habitual long-term exercise and acute bouts of physical exertion. Such enhancements are manifested in sharpened attentional focus, rigorous logical planning, and heightened decision-making efficiency, facilitating the management of complex academic demands (Liu et al., 2025). Mechanistically, PA transcends the physiological-cognitive boundary by remodeling neural networks through macro-structural and micro-functional pathways. At the macro-level, chronic exercise increases gray and white matter volume in critical regions, such as the hippocampus and cerebellum, providing a robust structural substrate for complex information processing. Concurrently, PA modulates activation within the dorsolateral prefrontal cortex and cingulate gyrus, strengthening functional connectivity and optimizing neural network topologies associated with EF (Cai and Zhang, 2019). At the microscopic level, these cognitive dividends are driven by an enriched biochemical milieu. PA triggers the synthesis of brain-derived neurotrophic factor (BDNF), which provides essential neurotrophic support for synaptic plasticity and neuronal survival. Furthermore, PA-induced increases in cerebral blood flow enhance nutrient delivery and energy metabolism within neural cells (Mandolesi et al., 2018). Given the dual physiological and psychological pressures encountered by the collegiate population, investigating the protective effects of PA on EF is imperative. Supported by these structural and biochemical insights, physical activity represents a pivotal intervention for elevating cognitive performance. Consequently, this study proposes Hypothesis H1: Physical activity positively predicts the development of executive function in college students.

Beyond physiological optimization, the enhancement of EF through PA entails a profound mechanism of psychological regulation: it effectively mitigates the maladaptive depletion of cognitive resources by alleviating anxiety. Anxiety, fundamentally, is a distressing subjective experience—characterized by a confluence of tension, apprehension, and worry—that manifests during encounters with uncertainty or perceived threats, typically accompanied by defensive autonomic nervous system activation (Hartley and Phelps, 2012). The cognitive interference of this emotional state is elucidated by Processing Efficiency Theory, which posits that elevated anxiety levels induce a cascade of task-irrelevant cognitions, such as ruminative worrying. These intrusive thoughts preemptively occupy working memory resources essential for task execution. Consequently, anxious individuals are compelled to exert disproportionately high mental effort to maintain performance parity with their less-anxious counterparts, leading to an inefficient cognitive cycle. This psychological burden is pervasive within contemporary academic environments. Empirical surveys suggest that approximately 23.3% of Chinese university students experience clinically significant anxiety (Li B. et al., 2024), a factor that substantially impedes the operational efficiency of EF. Neuroscientific evidence further underscores the physiological underpinnings of this phenomenon, demonstrating that anxiety significantly dysregulates activation within the dorsal and ventral prefrontal cortices, as well as the anterior cingulate cortex (Koay and Van Meter, 2023)—the critical neural substrates of cognitive control. Given that PA is known to modulate these neural circuits and stabilize emotional oscillations, anxiety may serve as a functional bridge between exercise and cognition. Building upon this theoretical framework, this study proposes Hypothesis H2: Anxiety mediates the relationship between physical activity and the development of executive function in college students.

Beyond immediate physiological benefits, the role of PA in optimizing sleep quality (SQ) is paramount. Enhanced sleep not only facilitates essential systemic repair but also establishes a robust physiological substrate—a “cognitive reservoir—necessary for the efficient operation of EF. Sleep is an active, multifaceted neurophysiological process that critically regulates cognitive performance, physical recuperation, and overall psychosomatic equilibrium (Rushworth et al., 2026). Consequently, as a fundamental determinant of EF development, the caliber of sleep quality directly influences the cerebral efficiency of information processing (Tai et al., 2022). However, this cognitive cornerstone is increasingly compromised within academic environments. Empirical data suggest that approximately 16.7% of Chinese university students suffer from poor sleep quality (Li B. et al., 2024), a deficit that frequently precipitates a precipitous decline in cognitive control. From a functional perspective, restorative sleep is indispensable for maintaining the integrity of EF. This is evidenced in behavioral tasks by significantly reduced latency and increased accuracy—benefits that are particularly pronounced in the domains of inhibitory control and cognitive flexibility. Although the magnitude of sleep’s impact on cognition may fluctuate across the lifespan, optimizing SQ remains a powerful intervention for bolstering neural plasticity and navigating cognitive challenges during critical developmental windows (Rushworth et al., 2026). Given that high-quality sleep serves as a vital physiological foundation for cognitive health, it may represent a pivotal hub through which PA is converted into cognitive gains. Accordingly, this study proposes Hypothesis H3: Sleep quality mediates the relationship between physical activity and the development of executive function in college students.

Investigations into the mechanistic underpinnings of psycho-physiological health demonstrate that sleep and anxiety are not isolated constructs; rather, they function as both antecedents and consequences within a reciprocal regulatory network of cognition. From a neurobiological perspective, EF is inextricably linked to the activation of the prefrontal cortex (PFC). As the primary hub for high-level decision-making and inhibitory control, the PFC is exceptionally susceptible to nocturnal sleep status. In states of sleep deprivation, the functional development and operational integrity of the PFC are compromised. This depletion of cognitive resources diminishes the brain’s capacity to buffer negative affect, rendering individuals increasingly vulnerable to anxiety under stressful conditions. Longitudinal evidence identifies chronic sleep deficits as a robust predictor of future anxiety levels (Tai et al., 2022). The transition from this depletion to a deleterious cycle is likely driven by the anxiety-induced hyperarousal of the autonomic nervous system. Individuals in anxious states frequently undergo a cascade of physiological responses, including tachycardia and respiratory dysregulation. These autonomic fluctuations facilitate sleep fragmentation, significantly increasing sleep latency. Furthermore, under conditions of uncertainty, high-anxiety individuals often exhibit a pronounced probability bias (PB), characterized by the maladaptive magnification of potential risks. This cognitive distortion persistently occupies finite attentional resources; even during nocturnal periods, the brain remains unable to transition into a restorative state due to this cognitive overload. Such resource scarcity becomes manifest during complex task execution. When sleep deficiency erodes foundational neural energy, the cognitive redundancy triggered by anxiety further crowds out the functional space required for EF. Consequently, on the decision-making spectrum, individuals often display a marked tendency toward risk aversion, adopting excessively conservative strategies (Hartley and Phelps, 2012). Given this logic of bidirectional attrition, exploring whether physical activity can optimize this pathway is imperative. Accordingly, this study proposes Hypothesis H4: Anxiety and sleep quality play a chain-mediating role in the process through which physical activity influences the development of executive functions in college students.

**Figure 1:**
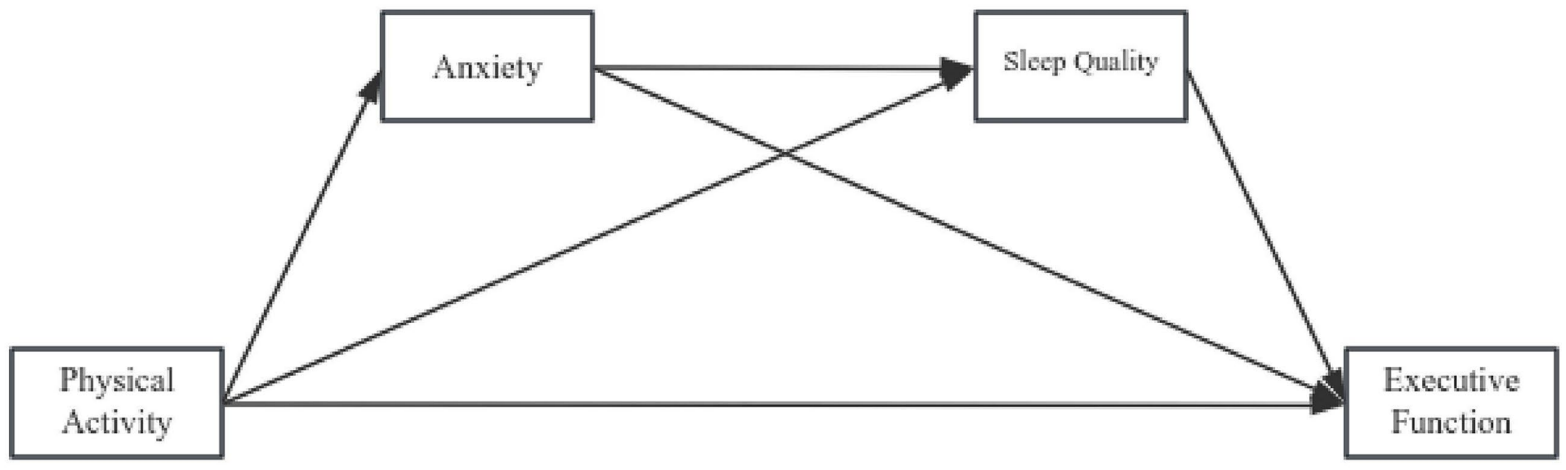
Chain mediation model.

In summary, although prior research has substantiated the positive influence of PA on EF and independently examined the cognitive impacts of sleep and anxiety, contemporary evidence remains largely compartmentalized. Specifically, the existing literature lacks a cohesive, systematic framework that integrates anxiety and sleep quality as sequential mediating factors. Within the university context, pervasive academic stressors and irregular routines frequently attenuate the cognitive dividends typically derived from exercise. This disparity underscores a pivotal scientific inquiry: when designing exercise interventions, how can physiological restoration and psychological buffering be synergistically balanced to maximize cognitive gains? Presently, both a comprehensive theoretical architecture and robust empirical validation for this integration are lacking. Motivated by this identified research lacuna, the present study seeks to move beyond the constraints of previous unilinear correlations. By constructing a chain mediation model incorporating anxiety and sleep quality, we aim to elucidate the underlying pathways through which physical exercise modulates executive function among college students, as illustrated in Figure 1. Ultimately, this research endeavors to provide targeted empirical evidence to refine and optimize cognitive health intervention strategies tailored for the university population.

## 2 Participants and methods

### 2.1 Participants

Data collection for this study commenced in December 2025. To optimize sample representativeness alongside logistical feasibility, we employed a multi-stage cluster convenience sampling strategy rather than simple random sampling. Leveraging established inter-institutional cooperative networks, a cross-sectional survey was conducted across six representative Chinese provinces (including Hunan, Sichuan, Shaanxi, Shanxi, Anhui, etc.). This pragmatic design, facilitated by local school administrators, ensured high response efficiency and broad geographic coverage. At the operational level, the academic class served as the primary sampling unit. Upon securing institutional cooperation, all classes meeting the predetermined inclusion criteria were recruited. Participants completed standardized psychological instruments under the guidance of trained researchers to ensure data integrity.

Prior to formal administration, the protocol received rigorous ethical approval from the Institutional Review Board (IRB) of the authors’ home institution. To align with contemporary digital data collection paradigms, questionnaires were administered via secure online platforms and distributed through institutional communication channels. The informed consent interface explicitly detailed the research objectives, the principle of strict anonymity, and robust privacy protection measures. Participants were required to provide active electronic consent before proceeding to the survey items. Furthermore, participant autonomy was prioritized, with the explicit right to unconditional withdrawal at any stage of the process.

Stringent quality control measures were implemented to mitigate the inclusion of low-quality or aberrant responses. A pilot study established a normative response duration; consequently, questionnaires completed in less than 3 min or exceeding 9 min were excluded as outliers. Additionally, protocols involving invariant response patterns (straight-lining) or substantial missing data were discarded. The final analytic sample comprised 2,033 respondents (19.0 ± 1.1), consisting of 882 males (43.4%) and 1,151 females (56.6%). Among them, 394 (19.4%) were only children, and 1,639 (80.6%) were not only children. Detailed demographic information is presented in Table 1. Through this series of rigorous procedures, a solid data foundation has been established for the subsequent mechanism analysis.

**Table 1:** Demographic profile of participants.

Features	Category	*N*	Percentage (%)
Gender	Male	882	43.4
	Female	1,151	56.6
Only-child status	Yes	394	19.4
	No	1,639	80.6
Father’s education level	Unknown	114	5.6
	Primary school or below	415	20.4
	High school	1,288	63.4
	University	201	9.9
	Postgraduate	15	0.7
Mother’s education level	Unknown	130	6.4
	Primary school or below	552	27.2
	High school	1,156	56.9
	University	183	9.0
	Postgraduate	12	0.6

### 2.2 Instruments

#### 2.2.1 Physical activity

PA was assessed using the Single-Item Measure (SIM) (Milton et al., 2011). This method has demonstrated superior recall effectiveness for the past week compared to the past month and exhibits robust test–retest reliability among adult populations (O'Halloran et al., 2022). It has been validated as an effective indicator of whether an individual meets the physical activity standards recommended by the World Health Organization (WHO) (Bauman and Richards, 2022). Following recent academic protocols (Wang et al., 2025a; Jia et al., 2025; Wang et al., 2025b), the current study measured physical activity levels with the following question: “Please recall how many days in the past week you engaged in at least 30 min of moderate-to-vigorous physical activity,” with responses ranging from 0 to 7 days.

#### 2.2.2 Generalized anxiety disorder-2 (GAD-2)

Developed by Spitzer et al., this scale is used to screen and evaluate generalized anxiety disorder (Skapinakis, 2007). It utilizes a 4-point Likert scale (0 = Not at all, 1 = Several days, 2 = More than half the days, 3 = Nearly every day), where higher scores indicate higher levels of anxiety. The scale focuses on the core experiences of anxiety with 2 items and a maximum score of 6. In this study, the Cronbach’s *α* coefficient for this scale was 0.85.

#### 2.2.3 Single-Item Sleep Quality Scale (SQS)

The Single-Item Sleep Quality Scale (SQS), developed by Snyder et al., was used to assess participants’ overall sleep quality during the past week and has been validated as an efficient alternative to multi-item sleep quality instruments (Snyder et al., 2018). Consistent with the original scale format, participants rated their sleep quality on a 10-point Likert scale ranging from 1 (“terrible”) to 10 (“excellent”), with higher scores indicating better perceived sleep quality. Structurally, the SQS functions as a single global item rather than a multidimensional subscale measure. Respondents are asked to consider several key aspects of their recent sleep experience, including sleep duration, difficulty falling asleep, frequency of nocturnal awakenings, frequency of early morning awakenings, and the extent to which they felt refreshed after sleep, before providing an overall evaluation of their sleep quality.

#### 2.2.4 Adolescent Executive Function Scale

Developed by Huang et al., this scale evaluates an individual’s EF (Huang et al., 2014). It uses a 3-point Likert scale (1 = Never, 2 = Sometimes, 3 = Often), where higher scores indicate poorer EF and lower scores indicate better performance. The scale comprises 21 items across three dimensions: inhibitory control (IC), working memory (WM), and cognitive flexibility (CF), with a maximum score of 63. In this study, the Cronbach’s *α* coefficient was 0.87.

### 2.3 Covariate control

To isolate the potential confounding effects of extraneous variables on the complex interrelationships between physical activity, anxiety, sleep quality, and executive function, this study incorporated several demographic covariates into the analytical model. Specifically, variables including gender, age, academic grade, parental educational attainment, and only-child status were strictly controlled. This approach was designed to account for variance arising from biological sex differences, neurodevelopmental stages, fluctuating academic demands, socioeconomic background, and domestic structure. By adjusting for these specific covariates, the present study aims to more precisely delineate the mechanistic pathways through which physical activity modulates executive function—mediated by anxiety and sleep quality—thereby ensuring the internal validity and empirical rigor of the findings.

### 2.4 Data analysis

Common method bias testing (CMB), descriptive statistics, and correlation analyses were performed using SPSS 25.0. CMB was assessed via factor analysis, and correlations between variables were tested using Pearson’s correlation analysis. The chain mediation effect was examined using the SPSS PROCESS macro (Model 6) and the Bootstrap method. The significance level was set at *α* = 0.05. During parameter estimation, 5,000 resamples were performed using the bias-corrected Bootstrap method, and 95% bias-corrected confidence intervals were generated accordingly. An effect was considered statistically significant when its confidence interval did not include zero.

## 3 Results

### 3.1 Common method bias test

The results of Harman’s single-factor test revealed 14 factors with eigenvalues >1. The variance explained by the first factor was 37.98%, which is below the critical threshold of 40%. This indicates that there is no serious issue of common method bias in the present study.

### 3.2 Correlation analysis

Correlation analysis were conducted among physical activity, anxiety, sleep quality, and executive function in college students. As shown in Table 2, the correlations between the four variables were all statistically significant. Specifically, PA was negatively correlated with anxiety (*r* = −0.095) and positively correlated with SQ and EF (*r* = 0.132 and *r* = 0.116). Anxiety showed negative correlations with SQ and EF (*r* = −0.360 and *r* = −0.447). SQ was positively correlated with EF (*r* = 0.294). All the aforementioned differences were statistically significant.

**Table 2:** Correlation matrix of variables.

Variables	*M* ± *SD*	PA	Anxiety	SQ	IC	WM	CF	EF
PA	3.33 ± 1.99	–						
Anxiety	3.31 ± 1.20	−0.095**	–					
SQ	7.04 ± 1.92	0.132**	−0.360**	–				
IC	6.96 ± 1.29	0.102**	−0.366**	0.231**	–			
WM	6.52 ± 1.45	0.088**	−0.330**	0.236**	0.525**	–		
CF	8.87 ± 1.81	0.102**	−0.420**	0.268**	0.552**	0.583**	–	
EF	22.35 ± 3.82	0.116**	−0.447**	0.294**	0.797**	0.880**	0.832**	–

**p < 0.01.

### 3.3 Chain mediation effect test

A chain mediation analysis was conducted with PA as the independent variable, EF as the dependent variable, and anxiety and SQ as the mediating variables. Gender, age, parental education level, grade, and only-child status were included as control variables.

As shown in Table 3, PA negatively predicted anxiety (*β* = −0.089, *t* = −3.973); anxiety, in turn, negatively predicted SQ (*β* = −0.350, *t* = −16.905). PA was found to positively predict SQ (*β* = 0.098, *t* = 4.678). Furthermore, SQ positively predicted EF (*β* = 0.151, *t* = 7.194), while anxiety negatively predicted EF (*β* = −0.383, *t* = −18.347). Finally, PA positively predicted EF (*β* = 0.053, *t* = 2.650). The 95% confidence intervals (CI) for all indirect effects did not contain zero, indicating that all mediating pathways were statistically significant.

**Table 3:** Regression analysis of relationships of variables for total executive function.

Dependent variables	Independent variables	*R*	*R^2^*	*F*	*β*	*t*	*p*
Anxiety		0.109	0.012	3.464			
	PA				−0.089	−3.973	<0.001
	Gender				0.028	1.206	0.228
	Age				−0.011	−0.382	0.703
	Father’s education level				−0.028	−0.953	0.341
	Mother’s education level				−0.022	−0.756	0.450
	Grade				0.013	0.448	0.655
	Only-child status				−0.003	−0.136	0.892
SQ		0.380	0.144	42.624			
	PA				0.098	4.678	<0.001
	Anxiety				−0.350	−16.905	<0.001
	Gender				0.023	1.116	0.265
	Age				0.003	0.119	0.905
	Father’s education level				0.019	0.679	0.498
	Mother’s education level				0.009	0.314	0.754
	Grade				−0.057	−2.146	0.032
	Only-child status				0.001	0.043	0.965
EF		0.486	0.237	69.638			
	PA				0.053	2.650	0.008
	Anxiety				−0.383	−18.347	<0.001
	SQ				0.151	7.194	<0.001
	Gender				−0.060	−2.957	0.003
	Age				0.039	1.547	0.122
	Father’s education level				0.036	1.396	0.163
	Mother’s education level				0.032	1.220	0.223
	Grade				0.044	1.760	0.079
	Only-child status				0.029	1.459	0.145

To further capture the fine-grained differences among EF sub-dimensions, additional path decomposition analyses were conducted separately for IC, WM, and CF (see Tables 4–10). The results revealed nuanced structural differences across these three components. Specifically, the direct predictive effect of PA was most pronounced for IC, with the direct pathway accounting for 55.67% of the total effect in this model. For CF, the psychological regulatory pathway through anxiety reduction showed the strongest explanatory contribution, accounting for 36.26% of the total effect. In addition, although the chain mediation pathway of “PA → anxiety reduction → sleep restoration → cognitive benefit” remained significant across all EF indicators, its relative contribution was highest in the WM model, accounting for 4.82% of the total effect.

**Table 4:** Regression analysis of relationships of variables for inhibitory control.

Dependent variables	Independent variables	*R*	*R^2^*	*F*	*β*	*t*	*p*
Anxiety		0.109	0.012	3.464			
	PA				−0.089	−3.973	<0.001
	Gender				0.028	1.206	0.228
	Age				−0.011	−0.382	0.703
	Father’s education level				−0.028	−0.953	0.341
	Mother’s education level				−0.022	−0.756	0.450
	Grade				0.013	0.448	0.655
	Only-child status				−0.003	−0.136	0.892
SQ		0.380	0.144	42.624			
	PA				0.098	4.678	<0.001
	Anxiety				−0.350	−16.905	<0.001
	Gender				0.023	1.116	0.265
	Age				0.003	0.119	0.905
	Father’s education level				0.019	0.679	0.498
	Mother’s education level				0.009	0.314	0.754
	Grade				−0.057	−2.146	0.032
	Only-child status				0.001	0.043	0.965
IC		0.393	0.154	41.009			
	PA				0.054	2.592	0.010
	Anxiety				−0.319	−14.537	<0.001
	SQ				0.105	4.762	<0.001
	Gender				−0.018	−0.413	0.680
	Age				0.045	1.941	0.052
	Father’s education level				0.061	1.591	0.112
	Mother’s education level				0.038	1.041	0.298
	Grade				−0.053	−1.221	0.222
	Only-child status				0.031	0.587	0.557

**Table 5:** Regression analysis of relationships of variables for working memory.

Dependent variables	Independent variables	*R*	*R^2^*	*F*	*β*	*t*	*p*
Anxiety		0.109	0.012	3.464			
	PA				−0.089	−3.973	<0.001
	Gender				0.028	1.206	0.228
	Age				−0.011	−0.382	0.703
	Father’s education level				−0.028	−0.953	0.341
	Mother’s education level				−0.022	−0.756	0.450
	Grade				0.013	0.448	0.655
	Only-child status				−0.003	−0.136	0.892
SQ		0.380	0.144	42.624			
	PA				0.098	4.678	<0.001
	Anxiety				−0.350	−16.905	<0.001
	Gender				0.023	1.116	0.265
	Age				0.003	0.119	0.905
	Father’s education level				0.019	0.679	0.498
	Mother’s education level				0.009	0.314	0.754
	Grade				−0.057	−2.146	0.032
	Only-child status				0.001	0.043	0.965
WM		0.382	0.146	38.326			
	PA				0.041	1.918	0.055
	Anxiety				−0.273	−12.372	<0.001
	SQ				0.136	6.115	<0.001
	Gender				−0.108	−2.491	0.013
	Age				0.045	1.899	0.058
	Father’s education level				0.070	1.819	0.069
	Mother’s education level				0.033	0.878	0.380
	Grade				0.110	2.504	0.012
	Only-child status				0.099	1.854	0.064

**Table 6:** Regression analysis of relationships of variables for cognitive flexibility.

Dependent variables	Independent variables	*R*	*R^2^*	*F*	*β*	*t*	*p*
Anxiety		0.109	0.012	3.464			
	PA				−0.089	−3.973	0.000
	Gender				0.028	1.206	0.228
	Age				−0.011	−0.382	0.703
	Father’s education level				−0.028	−0.953	0.341
	Mother’s education level				−0.022	−0.756	0.450
	Grade				0.013	0.448	0.655
	Only-child status				−0.003	−0.136	0.892
SQ		0.380	0.144	42.624			
	PA				0.098	4.678	0.000
	Anxiety				−0.350	−16.905	0.000
	Gender				0.023	1.116	0.265
	Age				0.003	0.119	0.905
	Father’s education level				0.019	0.679	0.498
	Mother’s education level				0.009	0.314	0.754
	Grade				−0.057	−2.146	0.032
	Only-child status				0.001	0.043	0.965
CF		0.454	0.206	58.431			
	PA				0.041	1.996	0.046
	Anxiety				−0.363	−17.078	0.000
	SQ				0.136	6.329	0.000
	Gender				−0.156	−3.755	0.000
	Age				0.005	0.200	0.841
	Father’s education level				0.008	0.211	0.833
	Mother’s education level				0.037	1.036	0.301
	Grade				0.104	2.459	0.014
	Only-child status				0.054	1.049	0.294

**Table 7:** Path and effect decomposition for total executive function.

Effect	Path	Effect size	Proportion of mediating effect	SE	95%CI	
					LB	UB
Direct effect	Physical activity → Executive function in college students	0.053	49.53%	0.020	0.014	0.092
Mediation effect	Physical activity → Anxiety → Executive function in college students	0.034	31.78%	0.009	0.017	0.051
Mediation effect	Physical activity → Sleep quality → Executive function in college students	0.015	14.02%	0.004	0.007	0.023
Mediation effect	Physical activity → Anxiety → Sleep quality → Executive function in college students	0.005	4.67%	0.002	0.002	0.008
Total effect	Physical activity → Executive function in college students	0.107	100%	0.022	0.063	0.150

**Table 8:** Path and effect decomposition for inhibitory control.

Effect	Path	Effect size	Proportion of mediating effect	SE	95%CI	
					LB	UB
Direct effect	Physical activity → Inhibitory control	0.054	55.67%	0.021	0.013	0.096
Mediation effect	Physical activity → Anxiety → Inhibitory control	0.029	29.90%	0.008	0.014	0.044
Mediation effect	Physical activity → Sleep quality → Inhibitory control	0.010	10.31%	0.004	0.004	0.018
Mediation effect	Physical activity → Anxiety → Sleep quality → Inhibitory control	0.003	3.09%	0.001	0.001	0.006
Total effect	Physical activity → Inhibitory control	0.097	100%	0.022	0.053	0.141

**Table 9:** Path and effect decomposition for working memory.

Effect	Path	Effect size	Proportion of mediating effect	SE	95%CI	
					LB	UB
Direct effect	Physical activity → Working memory	0.041	49.40%	0.021	−0.001	0.082
Mediation effect	Physical activity → Anxiety → Working memory	0.024	28.92%	0.007	0.012	0.037
Mediation effect	Physical activity → Sleep quality → Working memory	0.013	15.66%	0.004	0.006	0.022
Mediation effect	Physical activity → Anxiety → Sleep quality → Working memory	0.004	4.82%	0.001	0.002	0.007
Total effect	Physical activity → Working memory	0.083	100%	0.022	0.039	0.126

**Table 10:** Path and effect decomposition for cognitive flexibility.

Effect	Path	Effect size	Proportion of mediating effect	SE	95%CI	
					LB	UB
Direct effect	Physical activity → Cognitive flexibility	0.041	45.05%	0.020	0.001	0.081
Mediation effect	Physical activity → Anxiety → Cognitive flexibility	0.033	36.26%	0.008	0.017	0.049
Mediation effect	Physical activity → Sleep quality → Cognitive flexibility	0.013	14.29%	0.004	0.006	0.021
Mediation effect	Physical activity → Anxiety → Sleep quality → Cognitive flexibility	0.004	4.40%	0.001	0.002	0.007
Total effect	Physical activity → Cognitive flexibility	0.091	100%	0.022	0.047	0.135

**Figure 2:**
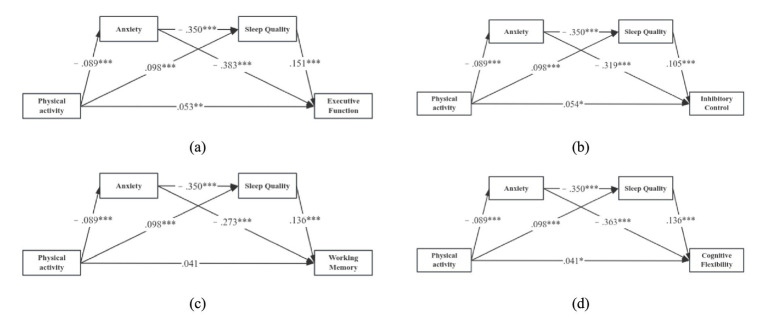
Mediation model of anxiety and sleep quality between physical activity and executive function and its sub-dimensions in college students **(a–d)**.

As shown in Table 7, the direct effect of physical exercise on college students’ executive function accounted for 49.53% of the total effect. The mediating effect of the pathway “Physical Activity → Anxiety → Executive Function” accounted for 31.78%, while the pathway “Physical Activity → Sleep Quality → Executive Function” accounted for 14.02%. Furthermore, the chain mediating effect of the pathway “Physical Activity → Anxiety → Sleep Quality → Executive Function” accounted for 4.67%. These results demonstrate that the following were all statistically supported: the mediating role of anxiety between physical activity and the development of executive function; the mediating role of sleep quality between physical activity and the development of executive function; and the chain mediating role of both anxiety and sleep quality in the relationship between physical activity and executive function among college students (Figure 2).

## 4 Discussion

The results of this study indicate that PA positively predicts the development of EF in college students, thereby validating Hypothesis H1 and maintaining consistency with previous findings (Dong et al., 2022). The path analysis results delineate a consistent pattern: the direct effect of physical exercise on EF accounts for 49.53% of the total variance. This finding empirically substantiates regular exercise as a primary pathway for cognitive optimization, aligning with the established theoretical conceptualization of exercise as a potent neuroprotective agent (Wen et al., 2023). From a neuroscientific perspective, the restorative impact of physical exertion on cognition is anchored in a robust biological substrate. Functional Magnetic Resonance Imaging (fMRI) data indicate that individuals with consistent exercise habits exhibit significantly higher activation within the prefrontal cortex (PFC) and the anterior cingulate cortex (ACC)—critical regions for high-level cognitive decision-making—during the execution of complex tasks (Salas-Gomez et al., 2020). This cognitive dividend typically emerges from the synergy of dual pathways. Specifically, acute exercise induces immediate physiological arousal, acting as a facilitator that acutely enhances the velocity and precision of cognitive processing. Concurrently, long-term habitual exercise fosters neuroplasticity, strengthening the structural and functional connectivity of core cognitive circuits (Tsuk et al., 2019; Tao et al., 2025). While the cognitive benefits of exercise demonstrate cross-age universality throughout the lifespan (Tian et al., 2023), these findings are particularly salient for the university population. In the face of prevalent sedentary behavior and emotional distress, increased physical activity has emerged as a widely endorsed intervention for bolstering cognitive control in this demographic (de Greeff et al., 2018; Li P. et al., 2024). Furthermore, the modality of exercise appears to be strategically significant. Compared to isolated strength training, activities requiring high levels of motor coordination—such as complex aerobic or skill-based tasks—more effectively pre-activate task-relevant neural networks. Due to the intense demands these activities place on the neuromuscular system, vestibular integration, and proprioception, they lead to a more pronounced enhancement of inhibitory control (Wen et al., 2023). This asymmetric pattern of baseline efficacy was further reflected in the differences observed across the direct pathways of EF sub-dimensions, with inhibitory control showing the highest effect coefficient (55.67%). The underlying mechanism of this pathway may be rooted in its frontal-lobe dependence. Regular physical activity can preferentially reshape neural networks and functional connectivity in core control regions, particularly the prefrontal cortex (Etnier and Chang, 2009). Because inhibitory control is highly dependent on these specific frontal circuits, it may be more sensitive to exercise-induced neural resource mobilization and physiological benefits (Tian et al., 2023; Van Der Niet et al., 2015). In conclusion, physical activity represents far more than a conventional means of fitness for college students. It functions as both a cognitive buffer against performance degradation and a critical restorative mechanism for mitigating the cognitive depletion induced by academic strain (Wang et al., 2024; Xiao et al., 2026). Collectively, this multidimensional protection establishes the cognitive foundation necessary for navigating rigorous academic challenges.

The findings of the present study confirm that anxiety serves as a pivotal mediator between PA and EF among college students. This empirical evidence provides robust validation for Hypothesis H2, aligning with prior scholarly observations that identify mental health as a critical determinant of cognitive performance (Ghrouz et al., 2019; Donnelly et al., 2024). From the perspective of cognitive resource allocation, anxiety functions as an emotional state driven by avoidance motivation. It acts as a persistent source of internal interference, inducing hyper-vigilance toward potential threats, which subsequently consumes the cognitive flexibility required for goal-directed task execution (Shields et al., 2016). According to Attentional Control Theory, anxiety significantly amplifies the automatic processing of threatening stimuli. This passive occupancy of cognitive channels monopolizes limited working memory capacity, thereby impeding task-relevant information processing (Sharp et al., 2015; Sheng et al., 2024). In academic and research settings, this mechanism is manifested through marked distractibility; students with elevated anxiety levels exhibit a sharp decline in selective and divided attention, becoming highly susceptible to task-irrelevant interference (Ajilchi and Nejati, 2017). This deleterious impact forms a complex cognitive-emotional feedback loop: anxiety impairs the neural capacity to inhibit irrelevant stimuli, leading to deficits in planning, inhibitory control, and cognitive switching (Ghrouz et al., 2019). Conversely, diminished EF weakens an individual’s ability to disengage from maladaptive thoughts, exacerbating worry and stress, and ultimately trapping the individual in a cycle of continuous resource depletion (Ajilchi and Nejati, 2017). PA offers a strategic intervention to break this maladaptive cycle (Pan et al., 2026; Yan et al., 2026a; Yang et al., 2026; Yan et al., 2026c; Yan et al., 2026b). Systematic reviews underscore that exercise is not only a preventive measure against psychiatric symptoms but also an effective modality for enhancing the overall quality of life in the university population (Donnelly et al., 2024). The primary mechanism involves the capacity of regular physical activity to attenuate the over-reactivity of the sympathetic nervous system and the hypothalamic–pituitary–adrenal (HPA) axis. By modulating the sensitivity of these stress-response systems, exercise stabilizes the psychological state (Anderson and Shivakumar, 2013). As exercise-induced anxiety reduction liberates previously occupied cognitive resources, students demonstrate a qualitative improvement in both the efficiency and accuracy of complex cognitive operations. With respect to differences among EF sub-dimensions, the buffering effect of PA against negative affect showed the strongest explanatory power in the cognitive flexibility pathway, accounting for 36.26% of the total effect. This pattern points to an important feature of cognitive vulnerability: anxiety-related hyperarousal may intensify cognitive rigidity and ruminative thinking. By alleviating this emotional load, PA may reduce the attentional resources consumed by irrelevant stimuli and restore the efficiency of attentional allocation required for rapid cognitive switching. The release of this capacity constitutes an important neural basis for maintaining a high level of cognitive flexibility (Dong et al., 2022; Malambo et al., 2022).

The findings of the present study identify a critical regulatory mechanism: SQ serves as a significant mediator between PA and EF in the university population. This conclusion provides robust empirical support for Hypothesis H3, corroborating the academic consensus that lifestyle factors modulate cognition through pathways of physiological restoration (Moussa-Chamari et al., 2024). As a highly plastic behavioral determinant, sleep functions as both the physiological substrate for maintaining peak cortical performance and a primary modulator of individual EF proficiency (Sen and Tai, 2023). When this physiological foundation is compromised, the structural integrity of cognitive processing undergoes significant degradation. Research indicates that both acute sleep deprivation and chronic declines in SQ result in marked deficits in sustained attention, psychomotor speed, and working memory. Furthermore, sleep insufficiency induces systematic decision-making biases during cognitive control tasks; specifically, sleep-deprived individuals often exhibit a reduced capacity for rational goal-orientation, relying instead on habitual and intuitive responses (Moussa-Chamari et al., 2024). These cognitive impairments correlate with observable neuroanatomical alterations. High-quality sleep is significantly positively associated with gray matter volume in several critical regions, including the orbitofrontal cortex (involved in high-order decision-making), the hippocampus (essential for memory consolidation), and the precentral gyrus (associated with motor regulation) (Tai et al., 2022). Conversely, chronic sleep deficiency may lead to localized structural atrophy in these areas. At the molecular level, sleep deprivation impairs the metabolic waste clearance efficiency of the glymphatic system and disrupts the stability of functional brain network connectivity, thereby undermining the neural architecture of EF (Sen and Tai, 2023). In addition, the physiological restorative effect of sleep and the chain mediation pathway showed their highest relative contributions in the working memory model, accounting for 15.66 and 4.82% of the total effect, respectively. The maintenance of working memory is highly dependent on the metabolic integrity of the prefrontal cortex, a region that is particularly sensitive to sleep deprivation. From a cognitive neuroscience perspective, high-quality sleep is essential for restoring the top-down control resources of the prefrontal cortex. Insufficient sleep may impair prefrontal recovery, thereby directly weakening the updating and maintenance of information in working memory (Zhang et al., 2023). It is through these physiological pathways that the cognitive benefits of PA are manifested. This study further confirms that PA-induced enhancement of EF is largely achieved by optimizing SQ and accelerating recovery from physiological fatigue—a process that secures the essential biological resources required for EF development (Anderson et al., 2009; Ji et al., 2022). For university students characterized by suboptimal physical health or prolonged academic strain, PA represents more than a psychological outlet; it constitutes a profound physiological restoration process. By improving the mediating variable of sleep, exercise does not merely alleviate affective distress but plays an active interventional role in preserving neural connectivity and promoting systemic recovery (Meng et al., 2025).

The findings ultimately elucidate a sophisticated regulatory loop: anxiety and SQ collectively constitute a significant chain mediating effect within the pathway through which PA influences the EF of college students. This discovery not only substantiates Hypothesis H4 but also delineates the intricate landscape of psycho-physiological interaction. As a non-pharmacological intervention, the benefits of PA do not represent a linear, unidimensional accumulation; rather, they reflect a multi-system synergistic response. Initially, by alleviating anxiety, exercise effectively reduces nocturnal autonomic nervous system arousal (Yang et al., 2025; Chen et al., 2025). Subsequently, PA-induced increases in cerebral blood flow and melatonin secretion further shorten sleep latency and optimize overall sleep architecture (Yang et al., 2025), establishing a robust physiological foundation for superior executive performance. In both clinical observations and the daily stress scenarios encountered by college students, anxiety and sleep appear as interdependent constructs (Klumpp et al., 2017). Within this population, anxiety functions as a substantial psychological stressor that disrupts sleep architecture and degrades SQ. High anxiety levels typically predict prolonged sleep onset latency, reduced total sleep duration, and diminished sleep efficiency (Chellappa and Aeschbach, 2022). Critically, sleep itself serves as a vital defense mechanism for maintaining emotional stability and preventing the exacerbation of anxiety. This bidirectional relationship is mediated through neuro-biochemical pathways: SQ modulates the intensity of the neural response to threatening stimuli. Once SQ declines, the top-down regulatory influence of the PFC over the limbic system weakens, leading to dysregulation within emotional circuits (Yang et al., 2025). This impairment manifests as a barrier to cognitive reappraisal; when performing cognitive restructuring tasks under stress, individuals with poor subjective SQ exhibit insufficient activation of the dorsal anterior cingulate cortex (dACC)—a region essential for cognitive control. Consequently, these individuals struggle to suppress anxiety by recalibrating cognitive strategies, thereby sustaining a maladaptive cycle of anxiety-insomnia (Klumpp et al., 2017). Given this complex interaction, the “Physical Activity → Anxiety → Sleep Quality → Executive Function” chain proposed in this study is of paramount importance. It underscores the profound value of PA in preserving cognitive resources beyond merely verifying the bidirectional linkage between anxiety and sleep. By utilizing regular exercise to buffer stress and stabilize psychological states, individuals can optimize SQ while simultaneously enhancing attentional focus and inhibitory control. This exercise-driven virtuous cycle of physiological function provides the necessary neural repair and resource security for EF development (Wang and Boros, 2021), ultimately facilitating a comprehensive advancement in cognitive performance.

An important point is that the findings should be interpreted within the broader bidirectional relationship between anxiety and sleep quality. The cross-sectional path framework used in this study specified a unidirectional mediating cascade and therefore did not capture the potential feedback pathway from sleep quality to anxiety. Despite this structural limitation, the significant empirical association (*β* = −0.350, t = −16.905) supports the specified “psychological-to-physiological” pathway, suggesting that cognitive overload and emotional instability may be important drivers of impaired sleep hygiene among college students. However, this statistically modeled direction does not negate the inherent reciprocal nature of psychophysiological health; rather, it provides a complementary perspective. In high-pressure academic environments, reducing emotional distress may represent a key entry point for disrupting the anxiety–insomnia cycle. By prioritizing emotional decompression, physical activity may weaken the cognitive interference that contributes to somatic arousal, thereby creating more favorable conditions for restorative sleep. Future studies should move beyond linear path models to more fully capture the reciprocal relationship between anxiety and sleep, as well as their combined depletion of EF. Ecological momentary assessment and cross-lagged regression models may help empirically disentangle the reverse “physiological-to-psychological” pathway and provide dynamic evidence on how sleep restoration buffers emotional stress. Although this study used a global measure of physical activity, future research on exercise and cognitive health should further consider the timing and type of physical activity (Ziereis and Jansen, 2015). On the one hand, the time window of a single exercise session may involve a dynamic dose–response balance. Brief acute exercise may be more likely to improve still-developing higher-order EF through transient neural facilitation, whereas longer-term habitual exercise may produce broader and more sustained improvements in working memory through accumulated neuroplastic adaptations (Benzing et al., 2016). On the other hand, the type of physical activity determines the extent to which cognitive resources are recruited (Daly et al., 2015). Compared with traditional aerobic exercise that relies more heavily on automated motor skills, cognitively engaging activities involving motor sequence learning, such as exergaming and coordination-based exercises, may generate greater cognitive benefits because they pre-activate core brain regions involved in EF. Therefore, future studies should not be limited to confirming the general benefits of exercise, but should further examine how specific exercise types and temporal doses can be precisely matched (Zhao et al., 2024), thereby informing individualized “exercise–cognition” intervention prescriptions for different populations.

In addition, the effects of demographic control variables in the model provide an important basis for understanding these cognitive mechanisms. The regression results showed that only-child status and parental educational attainment did not exert statistically significant direct effects on EF among college students (*p* > 0.05). The absence of a significant effect of parental education suggests that, during the university stage, individual cognitive ability and executive control may be more strongly shaped by lifestyle-related factors than by socioeconomic or family-of-origin background. These factors include structured physical activity, self-regulated sleep schedules, and timely anxiety management. Similarly, the non-significant effect of only-child status indicates that family structure may no longer serve as a decisive factor in cognitive development during early adulthood. Although biological sex showed a weak direct association with EF (*p* < 0.01), the overall empirical pattern suggests that behavioral habits may outweigh fixed family demographic characteristics in predicting cognitive performance among college students. These findings indicate that structured lifestyle interventions may produce stable cognitive benefits for university students regardless of their socioeconomic or family background.

Through a rigorous analysis of chain mediation effects, this study elucidates the central regulatory roles of anxiety and SQ in the relationship between PA and EF, establishing a cohesive conceptual framework. This mechanism progresses from external behavioral intervention to psychological modulation, transitions through physiological restoration, and ultimately culminates in cognitive optimization. Although the proportional contribution of this chain mediating effect within the total effect model may appear modest, its statistical significance underscores the inherent complexity of cognitive depletion mechanisms. This nuanced weighting serves as a critical reminder that EF enhancement is not a linear outcome of a singular intervention; rather, it is a multifaceted phenomenon that necessitates simultaneous management of the emotional-physiological cascade—specifically, the secondary decline in SQ induced by psychological distress. In contrast to previous literature focused on the direct PA–EF link, this research expands the theoretical horizon by incorporating the elimination of affective interference. By positioning anxiety as a pivotal mediator, the study explores how PA fortifies cognitive resilience through the dual mechanisms of psychological decompression and physiological sleep support. Leveraging a large-scale sample across six representative provinces, this robust path analysis offers profound practical insights: the cognitive gains derived from exercise transcend a rudimentary physiological stimulus, representing instead a sophisticated interplay of mind–body synergy. In essence, attenuated anxiety levels establish the psychological prerequisite for restorative sleep, while enhanced SQ provides the physiological substrate for the effective operation of executive processes. These findings have important implications for higher education administration and policy development. Universities should not regard physical education as an isolated elective component; rather, daily physical activity should be positioned as a foundational pillar of cognitive risk management. For policymakers, practical implementation requires structural curriculum reform, including allocating scheduled time for moderate-to-vigorous physical activity at the institutional level and improving access to recreational facilities. In addition, because anxiety management and sleep restoration were identified as key links in the cognitive transmission pathway, health interventions should not be implemented in isolation. Instead, physical fitness programs should be coordinated with existing campus psychological counseling services and sleep hygiene education, allowing physical education professionals, clinical counselors, and sleep health specialists to jointly design and deliver integrated courses and intervention programs. By defining physical activity as a preventive cognitive tool, rather than merely as a component of physical health, universities can develop a more comprehensive health promotion strategy to support students’ overall academic performance and long-term psychological resilience.

## 5 Limitations and future research directions

While the present study systematically elucidates the internal linkages between PA, anxiety, SQ, and EF through a validated chain mediation model, the rigor of scientific inquiry necessitates a critical reflection on several inherent constraints. A primary methodological consideration involves the cross-sectional nature of the research design. Although the hypothesized pathways were grounded in established theoretical frameworks, the reliance on single-point data collection precludes the ability to infer definitive temporal causality. To more dynamically capture the evolution of these interrelationships, future investigations should employ longitudinal tracking or experimental interventions, which would allow scholars to verify the causal directionality and developmental trajectories through which PA enhances cognitive performance.

Although this study further decomposed EF into three specific operational sub-dimensions, the reliance on self-reported instruments for assessing exercise, anxiety, sleep, and EF also presents opportunities for further refinement. While this approach is methodologically advantageous for securing large-scale samples, it remains susceptible to social desirability bias and retrospective memory distortion. To bolster measurement precision, subsequent research could integrate wearable physiological sensors—such as actigraphy or heart rate monitors—alongside objective cognitive task paradigms.

Synthesizing these multimodal data sources would significantly mitigate subjective bias and provide a more objective physiological substrate for the findings. Furthermore, the deconstruction of core variables warrants greater granularity in future analytical models. Additionally, the potential moderating roles of PA quality and quantity remain an open question. Whether the specific type, intensity, or frequency of physical exertion alters the strength of the mediational pathway requires further empirical scrutiny.

By reflecting on these boundaries, this study not only contextualizes the current findings but also charts a pragmatic course for subsequent inquiry: advancing the scientific understanding of collegiate cognitive health through more granular measurement and rigorous experimental designs.

## 6 Conclusion

This large-scale empirical investigation, involving 2,033 college students, provides a comprehensive analysis of the multidimensional mechanisms through which PA modulates EF. Transcending traditional unilinear causal inquiries, this research not only confirms the direct role of PA in cognitive enhancement but also employs sophisticated path analysis to delineate a mechanistic panorama extending from behavioral intervention to psychosomatic restoration.

The findings confirm a significant positive association between PA and EF among college students and further reveal important functional differentiation across EF sub-dimensions. PA should not be understood as a uniform cognitive stimulus; rather, it appears to operate through differentiated regulatory pathways. Specifically, PA directly promotes the improvement of inhibitory control, whereas its effects on cognitive flexibility and working memory seem to depend more strongly on the subsequent emotional buffering and sleep-restorative mechanisms triggered by exercise. The empirical evidence from both parallel and chain mediation pathways suggests that emotional stability and physiological restoration are not merely secondary by-products of exercise, but essential prerequisites for the optimization of higher-order cognitive functioning.

This psychosomatic synergy holds significant pedagogical and clinical implications. The value of PA lies in its dual capacity to expand cognitive reservoirs while simultaneously mitigating resource depletion. By attenuating anxiety, exercise minimizes the maladaptive consumption of cognitive resources induced by negative affective fluctuations. Concurrently, by improving SQ—the physiological substrate of neural health—exercise provides a critical window for metabolic clearance and resource consolidation. The convergence of these two forces facilitates a synergistic breakthrough in students’ executive capabilities.

As a cost-effective and high-utility intervention, PA serves as both a catalyst for academic achievement and a psychological buffer against emotional dysregulation in high-pressure environments. In conclusion, the role of physical education within higher education should transcend the traditional focus on physical fitness. Institutions should prioritize the efficacy of movement in shaping high-level cognitive functions and encourage students to regulate negative affect through active engagement in PA. By integrating exercise interventions, psychological counseling, and sleep hygiene management, a comprehensive optimization of mental health and cognitive proficiency can be achieved, establishing a robust psychosomatic defense for students as they navigate future academic and life challenges.

## Data Availability

The raw data supporting the conclusions of this article will be made available by the authors, without undue reservation.
